# Her2 assessment using quantitative reverse transcriptase polymerase chain reaction reliably identifies Her2 overexpression without amplification in breast cancer cases

**DOI:** 10.1186/s12967-017-1195-7

**Published:** 2017-05-01

**Authors:** Gabriele Zoppoli, Anna Garuti, Gabriella Cirmena, Ludovica Verdun di Cantogno, Cristina Botta, Maurizio Gallo, Domenico Ferraioli, Enrico Carminati, Paola Baccini, Monica Curto, Piero Fregatti, Edoardo Isnaldi, Michela Lia, Roberto Murialdo, Daniele Friedman, Anna Sapino, Alberto Ballestrero

**Affiliations:** 1 Department of Internal Medicine (Di.M.I.), University of Genoa and IRCCS AOU San Martino-IST, Viale Benedetto XV, 6, 16132 Genoa, Italy; 2 Department of Biomedical Sciences and Human Oncology, University of Turin, Turin, Italy; 3 Comprehensive Cancer Center Léon Bérard, Lyon, France; 4 Department of Pathology, University of Genoa and IRCCS AOU San Martino-IST, Genoa, Italy; 5 Department of Surgery, University of Genoa and IRCCS AOU San Martino-IST, Genoa, Italy

## Abstract

**Background:**

Immunohistochemistry (IHC) and fluorescent-in situ hybridization (FISH) are standard methods to assess human epidermal growth factor receptor 2 (HER2) status in breast cancer (BC) patients. Real-time quantitative polymerase-chain-reaction (qRT-PCR) is able to detect HER2 overexpression. Here we compared FISH, IHC, quantitative PCR (qPCR), and qRT-PCR to determine the concordance rates and evaluate their relative roles in HER2 determination.

**Patients and methods:**

We determined HER2 status in 153 BC patients, using IHC, FISH, Q-PCR and qRT-PCR. In discordant cases, we directly measured HER2 protein levels using Western blotting.

**Results:**

The overall agreement (OA) between FISH and Q-PCR was 94.1, with a k value of 0.87. Assuming FISH as the standard reference, Q-PCR showed an 86.1% sensitivity and a 99.0% specificity with a global accuracy of 91.6%. OA between FISH and qRT-PCR was 90.8% with a k value of 0.81. Of interest, the disagreement between FISH and qRT-PCR was mostly restricted to equivocal cases. HER2 protein analysis suggested that qRT-PCR correlates better than FISH with HER2 protein levels, particularly where FISH fails to provide conclusive results.

**Significance:**

qRT-PCR may outperform FISH in identifying patients overexpressing HER2 protein. Q-PCR cannot be used for HER2 status assessment, due to its suboptimal level of agreement with FISH. Both FISH and Q-PCR may be less accurate than qRT-PCR as surrogates of HER2 protein determination.

## Background

Human epidermal growth factor receptor 2 (HER2) is a predictive biomarker for therapeutic decisions in breast cancer.

Because direct HER2 protein assessment is not consistently reproducible in formalin-fixed tissues, a debate exists regarding the optimal surrogate tests for HER2 determination.

The American Society of Clinical Oncology/College of American Pathologists (ASCO/CAP) recommends IHC as a standard procedure for HER2 assessment, combined, in equivocal cases, with additional testing by in situ hybridization (ISH) assay with fluorescent (FISH) or chromogenic (CISH) probes [1, 2].

Moreover, ISH-based assessment is advocated for poor prognosis IHC +1 cases to avoid misclassification of such cases [3–5].

Although the IHC/ISH approach consents to classify the vast majority of HER2 positive tumors, these tissue-based tests are not devoid of interpretative issues and potential technical biases [6].

Combining IHC and ISH still yields to results falling on the equivocal area that could incorrectly subtract true positive cases from an effective anti-HER2 therapy. Furthermore, this analyses fail to identify a small but still clinically relevant proportion of patients that, although properly classified as non-HER2 amplified, overexpresses HER2 via non amplification-mediated mechanisms, and who may still benefit from treatment with trastuzumab [7–11].

Accordingly, there is room for improving current HER2 analysis methods with the use of alternative approaches.

The use of PCR-based tests in place of conventional methods is not routinely accepted, mostly because several studies failed to establish a consistently reproducible high level of agreement with “golden standard” tests [12].

Here we aimed to analyze HER2 RNA expression using quantitative real-time PCR (qRT-PCR), comparing the obtained results with PCR performed on DNA (Q-PCR) under rigorously controlled experimental conditions in 153 tumor samples. Moreover, we compared discrepancies between DNA and RNA measurement by Western blotting in a representative subset of cases. All samples underwent a systematic pre-test microscopy assessment to ensure sufficient tumor cellularity. Blinded independent operators performed FISH, IHC and the two PCR-based methods.

## Patients and methods

### Patients and study design

The study population included 153 patients with invasive ductal or lobular breast cancer retrospectively selected according to locally determined HER2 IHC and FISH results. We analyzed the above mentioned number of cases based on the assumption that we would need at least 152 samples to reach a kappa value of 0.95 for agreement between different methodologies, with 5% discordant cases.

The initial routine pathological examination, including HER2 IHC and FISH when necessary, was performed on formalin-fixed and paraffin-embedded (FFPE) tumor samples at the Pathology Unit of Genoa University.

We enriched this series in IHC HER2 positive (3+) patients to avoid the potential selection bias resulting from the high number of HER2-negative cases observed in routine practice. In fact, it has been demonstrated that the large prevalence of negative cases in not enriched series biases the comparison in favor of generating high concordances between assays [12].

In addition, we decided to include 22 cases (14% of the study population) classified as FISH equivocal, with the aim of a more statistically robust assessment of molecular tests performance in this small but clinically relevant subpopulation. To meet the selection criteria for inclusion in the present study, FISH equivocal cases required a confirmatory FISH re-counting of at least 60 invasive tumor cells.

After retrospective selection, all cases were tested for FISH, gene copy number and gene expression by independent, blinded operators. Molecular analyses were performed at the Department of Internal Medicine of Genoa University and FISH analysis was carried out at the Department of Biomedical Sciences and Human Oncology of Turin University.

Written informed consent for molecular analysis was obtained from all alive and traceable patients, in agreement with the Italian Law and the Declaration of Helsinki. Ethical approval of this retrospective study was granted by the IRCCS AOU San Martino-IST Research Ethics Committee (approval number P.R.01/13).

### Tissue specimens

Twelve tumor sections were obtained from the each FFPE block. The first and twelfth section (slides) of each sample were hematoxylin and eosin-stained (H&E-stained). These were examined by an experienced pathologist (P.B.) to determine the relative amount of tumor, benign epithelial and stromal cells, and to exclude the presence of in situ carcinomatous cells. Tumor sections with ≥70% of tumor cells were directly analyzed. Samples with a lower than 70% cellularity were subjected to macro- or micro-dissection (Laser Capture Microdissection, Histogene-Arcturus, CA). Sections #2 to #4 of each specimen (5 μm thick) were used for FISH analysis, whereas sections five to eleven (8 μm thick) were used for DNA and RNA extraction. Glass slides were stored at −80 °C until the extraction of nucleic acids.

### Immunohistochemistry and FISH analysis

For immunohistochemical HER2 determination, sections were incubated with the rabbit monoclonal primary antibody VENTANA anti-HER2/neu (4B5) (Ventana, Tucson, AZ, USA). Evaluation of IHC stains was performed by an experienced pathologist (P.B.), and scored according to the Manufacturer’s specifications [13]. FISH reaction was performed with PathVysion HER2 DNA Probe Kit (Abbott Molecular) according to the Manufacturer’s instructions. Analysis of HER2/CEP17 signals was done on ten areas selected by an experienced pathologist (L.V.C. and C.B.) and automatically acquired and read by the Imager MetaSystem (Zeiss) (http://www.metasystems-international.com), properly equipped, through PathVysionV2 classifier (FDA approved). Automatic reading of each case was verified with Isis software (Zeiss). Heterogeneous cases^3^ were counted on the Isis images. Cases were FISH scored according to ASCO/CAP 2013 guidelines and BICE AIOM/SIAPEC 2014 consensus recommendations [14, 15].

### Extraction of genomic DNA and total RNA

DNA was isolated using QIAamp DNA FFPE Tissue Kit (Qiagen GmbH, D-40724 Hilden) according to the Manufacturer’s protocol, and was therefore diluted in 60 μL elution buffer (ATE).

RNA was isolated using Paradise™ Reagent System (Arcturus) after incubation with proteinase K for 16 h at 56 °C. A Dnase I treatment step was included. RNA was diluted in 50 μL elution buffer (EB), according to the Manufacturer’s protocol.

DNA and RNA quantities were measured using two different methods, NanoDrop Spectrophotometer (ND-1000 Celbio, MI) and Qubit™ fluorimeter (Invitrogen, CA). Fluorimetric analysis was performed by Quant-iT dsDNA HS Assay Kit and Quant-iT™ Assay Kit (Invitrogen, CA) for, respectively, DNA and RNA.

### Quantitative PCR analysis

Quantitative PCR (Q-PCR) analyses for genes encoding HER2 and control Amyloid beta A4 precursor protein (APP, chromosomal location 21q21.3) were performed in 96-well plates and were carried out using the 7900HT Fast Real-Time PCR System (Applied Biosystems™, Foster City, CA, USA).

The sequences of the oligonucleotide primers and probes used for Q-PCR were derived from Lehman et al. [16].

Q-PCR conditions were as follows: PCR reactions were performed in 96-well plates with 7900HT fast PCR system (Applied Biosystems™—by Life Technologies™ Foster City, CA, USA). Reaction mixture for HER2, as well as APP reference gene, were set up in a 25 μL final volume containing up to 5 μL DNA (30 ng/μL), each 2.5 μL of the forward and reverse primers (final concentration: each 300 nM), 2.5 μL of the hybridization probes (final concentration 200 nM), and 12.5 μL of the Universal Master Mix (all reagents from Applied Biosystems™—by Life Technologies™ Foster City, CA, USA). The essays were started by denaturation for 2 min at 50 °C, 10 min at 95 °C and followed by 40 cycles of 95 °C for 15 s and 60 °C for 1 min. The HER2 amplified breast cancer cell line SKBR3 served as internal positive control.

### Quantitative real-time RT-PCR

To evaluate gene expression, the first-strand cDNA synthesis was performed from variable amounts (between 50 and 200 ng) of total RNA extracted with random hexamers by using High Capacity cDNA Archive Kit (Applied Biosystems™, Foster City, CA) in a final volume of 50 μL, according to the Manufacturer’s protocol.

HER2 and reference gene were preamplified using 12.5 μL of cDNA mixed with 25 μL of TaqMan PreAmp Master Mix and 12.5 μL of assay pool containing the mix of the two assays ID Hs00170433-ERBB2 and Hs99999902_m1-RPLP0 (Applied Biosystems™, Foster City, CA), 0.2X each. Preamplification allows for linear amplification of mRNA without significantly distorting relative mRNA levels [17].

The reaction was performed following the Manufacturer’s protocol with 14 cycles of amplification. The preamplification product was diluted 1:5. The qRT-PCR reactions were run for 2 min at 50 °C, 10 min at 95 °C, followed by 40 cycles of 15 s at 95 °C and 1 min at 60 °C. All qRT-PCR reactions, for HER2 as well as RPLP0 gene, were performed on the ABI 7900 HT system, and were measured in triplicate to ensure methodological reproducibility. This was considered adequate if within a maximum of 0.3 threshold PCR cycle (Ct) variation.

The qRT-PCR reactions were set up in 96-well plates, in a final volume of 20 μL containing up to 5 μL preamplified cDNA, 1 μL of TaqMan gene expression assay, 4 μL of molecular water, and 10 μL of the Universal Master Mix (all reagents from Applied Biosystems ™—by Life Technologies™ Foster City, CA, USA). Breast cancer cell line SKBR3, containing high levels of HER2, served as internal positive control.

### Calculation of HER2 gene copy number and expression

**Fig. 1:**
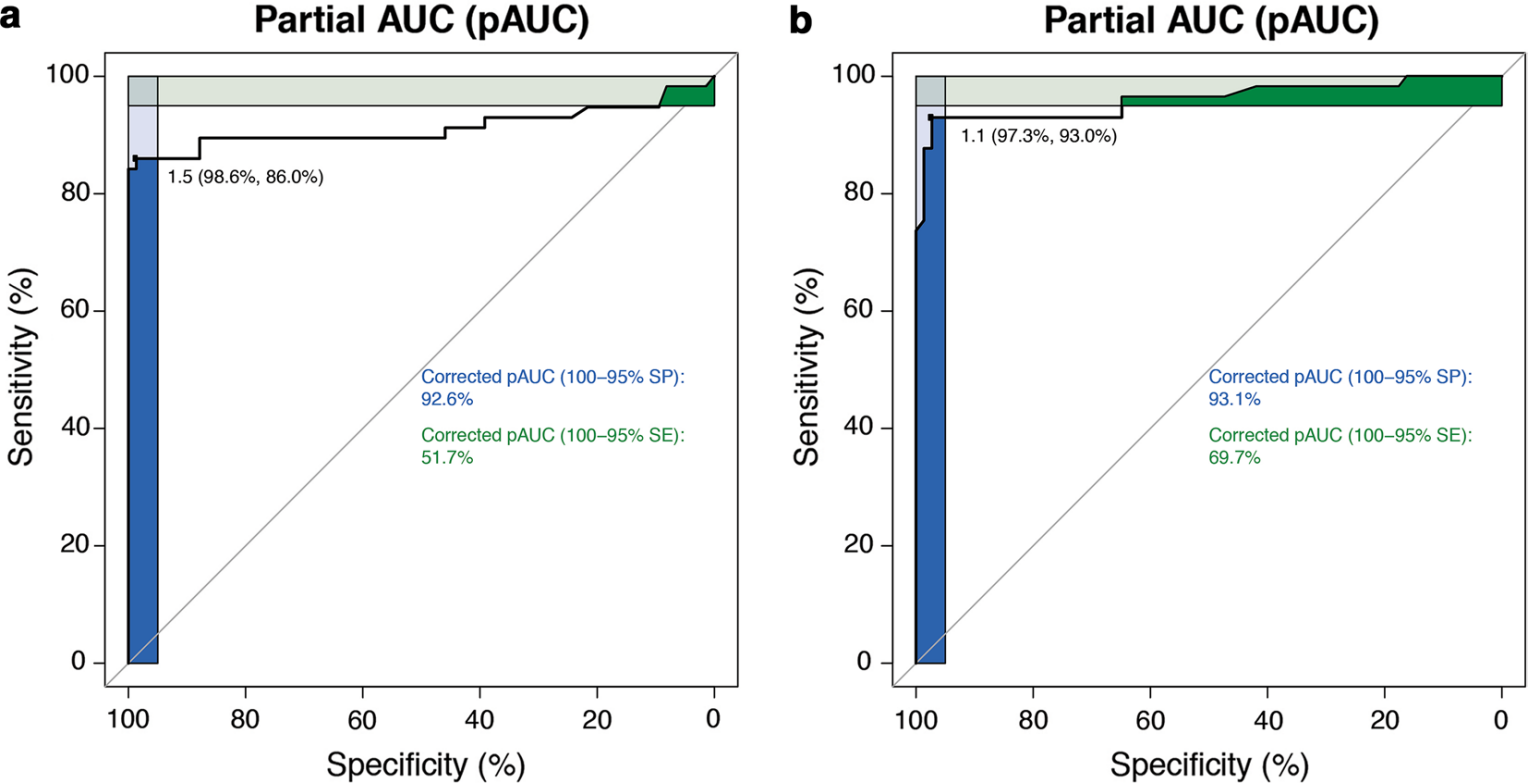
ROC curves for Q-PCR (**a**) and Q-RT-PCR (**b**) compared to FISH in non-equivocal cases. The *blue* and *green* squares represent the corrected partial areas under the curve (pAUC) for the graphic regions encompassing the 100–95% specificity and sensitivity areas, respectively. The value on the *topleft* corner of each* panel* indicates the optimal cutoff, with specificity and sensitivity in *parentheses*

In the real-time PCR-based quantitative analyses, initial DNA or RNA template concentration is correlated with the time at which the fluorescent signal crosses a threshold in the exponential phase of the polymerase chain reaction. Because this time is measured in terms of threshold PCR cycle (Ct), the initial template concentration can be derived from the Ct number. In each measurement, the normalization of DNA or RNA load by a housekeeping gene is required: gene reference (APP) to normalize the DNA and an housekeeping gene (RPLP0) to normalize the RNA. Amplification status of HER2 gene was determined on tumor DNA by Q-PCR. The APP gene was chosen as internal reference because its amplification efficiency was found to be similar to HER2 gene (data not shown) and infrequent copy number variations have been reported in breast cancer for this gene [16]. Amplification analysis is based on the ratio between the gene dosage measured in the tumor tissue sample and the corresponding measure in the normal human DNA chosen as calibrator (Human Genomic DNA, Takara Bio Europe/Clontech™, Saint Germain-en-Laye, France). Because this is a quantitative relative measure, the calculation was made according to the following formula: fold induction = 2^−[ΔΔCt],^ where ΔΔCt = [Ct HER2 (tumor sample) − Ct APP (tumor sample)] − [Ct HER2 (normal DNA) − Ct APP (normal DNA)]. Amplification was defined as a 2^−[ΔΔCt]^ result above the cut-off value of 1.5, determined in DNA samples from peripheral leukocytes of 25 normal donors and by the optimal value (defined as the threshold with the maximum sum of sensitivity and specificity) in the receiver-operating-characteristic (ROC) curves comparing Q-PCR with FISH status for the 121 non-equivocal cases of the present dataset (see Fig. 1a).

We determined HER2 gene expression on tumor RNA by quantitative RT-PCR technique (qRT-PCR). For this test, RPLP0 was adopted as endogenous reference gene, because it is reported as invariant in breast tissue and in preliminary experiments its amplification efficiency was found to be similar to HER2 gene (internal data and Lyng et al. [18]).

Relative quantification of HER2 gene expression was performed according to the 2^−ΔΔCt^ method. This algorithm is used to calculate the change in expression of a gene of interest relative to a calibrator sample chosen to represent 1× expression of the gene of interest. In the present analysis normal breast tissue (Human Mammary Gland Poli A+RNA Clontech, Mountain View, CA, USA) was adopted as calibrator.

The calculation of HER2 gene relative expression was performed according to the following formula: fold induction = 2^−[ΔΔCt],^ where ΔΔCt = [Ct HER2 (tumor sample) − Ct RPLP0 (tumor sample)] − [Ct HER2 (normal breast sample) − Ct RPLP0 (normal breast sample)]. Similar to Q-PCR, we determined the best cut-off to discriminate between HER2 positive and negative cases as a value = 1.1, by using the ROC curve method on the non-equivocal samples (see Fig. 1b).

We have to highlight that, in light of the variability of RNA and, partially, DNA degradation observed in FFPE tissue samples, the results of gene expression and copy number analysis can not be assumed as absolute measures. As a consequence, we transformed the obtained values into categorical variables, to compare them with results obtained by other analytical methods used to determine HER2 status [19, 20].

### Reconstitution in vitro experiments

To assess the dilution effect by not-HER2 amplified cells, tumor or not, on both Q-PCR and qRT-PCR methods, the levels of both HER2 copy number and expression were measured in serial dilutions of HER2 positive mammary carcinoma cell line SKBR3 with MCF10A cells, an immortalized HER2 negative breast tissue-derived cell line. In brief, different percentage dilutions of SKBR3:MCF10A were mixed, ranging from 100 to 5%, then the whole cell population was lysed and nucleic acids extracted. Copy number and expression analyses were performed on cell pellets according with the same methods used for tumor tissue sections, as described in the previous paragraphs. Results are reported as an average of three independent experiments.

### HER2 protein analysis (immunoblotting)

The FFPE tissue samples available for HER2 protein analysis were processed as follows. Four serial sections (about 100 mm^2^ and 8 μm thick) for each sample were placed in collection tubes (QIAGEN GmbH, Hilden). Protein extraction was performed using Qproteome FFPE Tissue Kit (QIAGEN GmbH, Hilden), according to the Manufacturer’s specifications. After we proceeded with the separation (25 μg for each sample) by SDS-PAGE (4–12%, Life Technologies™ Foster City, CA, USA). Proteins were transferred onto PVDF membrane (Immobilon-P, Millipore S.p.A., Vimodrone, Italy) and were detected using primary antibody for HER2 and Gamma Tubulin (Cell Signaling Technology, Inc. Danvers, MA 01923) and secondary antibodies horseradish peroxidase–conjugated anti-mouse or anti-rabbit (Cell Signaling Technology, Inc. Danvers, MA 01923). Enhanced chemiluminescence (Thermo Scientific Pierce ECL Western Blotting Substrate 3747 N Meridian Rd, Rockford, IL USA 61101) was detected by ChemiDoc (XRS+ System, Bio-Rad Laboratories Headquarters 1000 Alfred Nobel Drive Hercules, CA 94547).

HER2 protein quantity was calculated as HER2:tubulin optical density ratio assuming the ratio measured in MCF10 cell line as normal control.

### Statistical analysis

We performed concordance analysis (overall, positive and negative agreement) and the relative confidence intervals (Clopper-Pearson exact method) according to established definitions. The strength of agreement was evaluated using the Cohen’s unweighted kappa test. When assuming FISH as gold standard was appropriate sensitivity, specificity, positive and negative predictive values, likelihood ratios and accuracy, meant as the area under the ROC curve (AUC) according to the DeLong method, were calculated. The confidence intervals for likelihood ratios were obtained with the first method described in Using the first method described in Koopman, *Biometrics,* Vol. 40, No. 2 (Jun., 1984), pp. 513–517. Power calculations and statistical analyses were performed within the R environment for statistical computing, and the packages *statmod*, *gdata*, *gmodels*, *PropCIs*, *pROC*, and *psych* were used. All tests were two-tailed, and considered significant at a *P* value <0.05.

## Results

### Concordance between IHC and FISH

IHC and FISH testing results obtained in this study population are reported in Table 1. According to IHC score, 47 out of the 153 cases we included were classified as 3+, 49 as 2+, and 57 as either 1+ or 0. By FISH analysis, 51 patients (45 out of 47 IHC 3+ cases, and 6 out of 49 2+ cases) were HER2 amplified, while 102 were not (80 negative and 22 equivocal).

**Table 1:** Concordance between IHC versus FISH

IHC testing	FISH testing, *N* (%)	
	FISH positive	FISH negative
0	0 (0)	44 (100)
1+	0 (0)	13 (100)
2+	6 (12.2)	43 (87.8)
3+	45 (95.7)	2 (4.3)

*IHC* immunohistochemistry, *FISH* fluorescence in situ hybridization

IHC testing had a good degree of agreement with FISH, as classified by ASCO/CAP criteria 2013. The overall agreement (OA), calculated assuming IHC score 0, 1+ and 2+ as negative, and equivocal and negative cases together for FISH, was 92.2% (95% CI 86.7–95.9), average positive agreement (PA) was 88.5% (95% CI 80.7–93.9), and average negative agreement (NA) was 94.1% (95% CI 89.9–96.9). These data show discordance between IHC and FISH (7.8%, 95% CI 4.1–13.3) as in line with current results obtained by reference laboratories, with values around 5% [21].

### Dilution and amplification effects

Q-PCR and qRT-PCR give, respectively, the mean number of gene copies and the mean mRNA amount among all the cell populations present in any analyzed tumor tissue section.

**Fig. 2:**
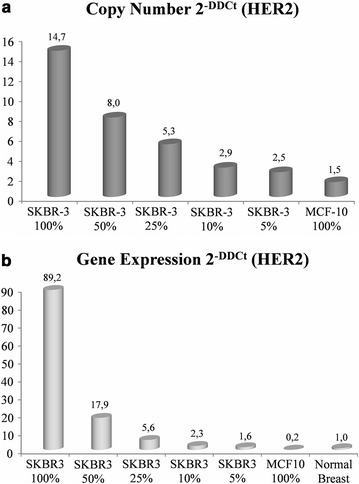
Dilution effect by not-HER2 amplified cells was analyzed in serial dilutions of HER2-positive SKBR3 cell line with HER2-negative MCF10A cell line. *Columns* represent the mean of three independent experiment performed by Q-PCR (**a**) and qRT-PCR (**b**). Copy number and gene expression were measured according to the 2^−[ΔΔCt]^ algorithm. SKBR3 is a mammary carcinoma cell line with a medium–high level of HER2 amplification. *HER2* human epidermal growth factor receptor 2, *Q-PCR* quantitative polymerase-chain-reaction, *qRT-PCR* quantitative reverse transcriptase polymerase-chain-reaction

The results of our in vitro experiments show that both tests were able to detect the signal in a wide range of SKBR3 to MCF10A ratios, from 100:0 to 5:95 (Fig. 2). The observed detection threshold of 5% in these in vitro conditions suggests that molecular tests have the ability to detect even very small fractions of positive cells among a whole cell population.

Overall, these results provide evidence against the hypothesis that the dilution effect could constitute a real bias in properly selected, microscopy assessed tumor specimens. Moreover, the absence of altered signal in the HER2-cell line and in normal breast tissue supports the conclusion that PCR methods do not detect HER2 signal when analyzed cell populations do not carry the molecular alteration.

### Concordance between FISH and Q-PCR

OA between FISH and Q-PCR, calculated considering FISH-equivocal cases as non-amplified, was 94.1% (95% CI 89.1–97.3%), PA was 91.6% (95% CI 84.6–96.1%), and NA was 95.5% (95% CI 91.6–97.9%). Agreement strength was very good with a k value of 0.87 (95% CI 0.79–0.95).

Assuming FISH as the reference standard, Q-PCR had a sensitivity = 86.0% (95% CI 74.2–93.7%), specificity = 99.0% (95% CI 94.3–99.9%), positive predictive value (PV) = 98.0% (95% CI 89.3–99.9%), negative PV = 92.2% (95% CI 85.3–96.6%), positive likelihood ratio = 82.5 (95% CI 11.7–581.5). Accuracy of Q-PCR in comparison, measured as the area under the ROC curve (AUC) with FISH was 91.6% (96% CI 85.4–97.9%).

### Concordance between FISH and qRT-PCR

FISH and qRT-PCR determine HER2 status at two different molecular levels (DNA and RNA). Hence, we only compared them by calculating agreement measures [22]. In our case series OA, calculated considering FISH-equivocal cases as non-amplified, was 90.8% (95% CI 85.1–94.9%), PA was 88.3% (95% CI 81.2–93.5%), and NA was 92.5% (95% CI 87.7–95.8%). The strength of agreement was very good, with a k value of 0.81 (95% CI 0.71–0.90).

### Analysis of the disagreement

Data reported in Table 2 show an increase of the overall agreement between mRNA expression (qRT-PCR) and both FISH and Q-PCR when the 22 equivocal cases are excluded from the analysis (95.4 vs. 90.8% for the comparison with FISH, and 94.7 vs. 90.2% for the comparison with Q-PCR). No such improvement is observed when the agreement between Q-PCR and FISH is assessed with or without the equivocal cases categorized as negative. We therefore tested the hypothesis that q-RT-PCR may actually recapitulate better HER2 protein overexpression than FISH in equivocal cases, by measuring HER2 protein levels in representative tumor samples.

**Table 2:** Overall agreement in patient subgroups: all cases versus not equivocal only

Comparisons	Overall agreement (95% CI)	
	All cases (153)	Non equivocal only (124)
FISH vs DNA copy number	94.10 (89.10–97.30)	97.58 (93.13–99.17)
FISH vs mRNA expression	90.80 (85.70–94.90)	95.40 (94.31–99.56)
DNA copy number vs mRNA expression	90.20 (84.46–93.97)	94.70 (90.91–98.27)

*CI* confidence interval, *FISH* fluorescence in situ hybridization, *DNA* deoxyribonucleic acid, *mRNA* messenger ribonucleic acid

In particular, we measured HER2 protein levels in six equivocal cases and in two remarkable HER2 non-amplified, mRNA overexpressing cases. Table 3 shows the results of Western blot analysis in which the amount of HER2 protein is normalized to the structural protein γ-tubulin. HER2 protein levels were low or undetectable (“negative” blots) in both the HER2-MCF10 control cell line and in three negative control patients, and high in both the HER2 positive SKBR3 cell line and in one positive control patient as assessed by FISH, Q-PCR and qRT-PCR (“positive blots”). Protein quantitation was in complete agreement with HER2 mRNA in the six FISH equivocal cases and in the two cases with mRNA overexpression with no evidence of amplification.

**Table 3:** Analysis of HER2 protein levels in a patient subset

Patient or control	IHC	FISH	Copy number	mRNA	Protein expression
MCF10	Neg	Neg	Neg	Neg	Neg
SKBR3	Pos	Pos	Pos	Pos	Pos
p1	Neg	Neg	Neg	Neg	Neg
p2	Neg	Neg	Neg	Neg	Neg
p3	Equivocal (2+)	Neg	Neg	Neg	Neg
p4	Pos	Pos	Pos	Pos	Pos
p5	Equivocal (2+)	Equivocal	Neg	Neg	Neg
p6	Equivocal (2+)	Equivocal	Neg	Neg	Neg
p7	Equivocal (2+)	Equivocal	Neg	Neg	Neg
p8	Neg	Equivocal	Neg	Pos	Pos
p9	Equivocal (2+)	Equivocal	Neg	Pos	Pos
p10	Equivocal (2+)	Equivocal	Neg	Pos	Pos
p11	Equivocal (2+)	Neg	Neg	Pos	Pos
p12	Equivocal (2+)	Neg	Neg	Pos	Pos

*HER2* human epidermal growth factor receptor 2, *IHC* immunohistochemistry, *FISH* fluorescence in situ hybridization, *mRNA* messenger ribonucleic acid, *neg* negative, *pos* positive

## Discussion

Molecular tests for HER2 status determination at DNA or RNA level have been widely studied for their potential advantages over FISH.

FISH and IHC can be time-consuming, expensive, and cumbersome for screening multiple samples over a short time. Moreover, these methods are difficult to standardize across laboratories [11, 23].

PCR-based methods may potentially improve the simplicity or accuracy of HER2 testing and have several advantages over current methods; they are quantitative by definition, do not require extensive training for their interpretation, they are not subject to interobserver variability, and can be standardized, automated and performed on small samples [24, 25].

Data from published studies comparing these different analytical methods are conflicting. At least nine publications compared DNA copy number measured by PCR with in situ methods, IHC/FISH-CISH. In the past different authors reported an insufficient concordance level [26–32] between Q-PCR and IHC/FISH or a clinically unacceptable high false negative rate [33], whereas other studies showed complete concordance [34, 35]. Particularly, a recent large study performed by Koudelova et al. showed that Q-PCR method reached a highly sensitive and specificity on the basis of comparison with FISH data (94.2 and 100%, respectively) and IHC data (95.1 and 99.1%). Furthermore the overall concordance of the FISH and Q-PCR results is 97.6% [36]. Our experience is in line with the observation of a high specificity at a cost of a slightly lower sensitivity of Q-PCR over FISH for HER2 status determination.

Several studies have also compared qRT-PCR based gene expression assessment with IHC/FISH. Two of them suggested a good agreement between FISH or IHC and gene expression analysis performed by real-time PCR method, but they were conducted on small patient series [37, 38]. Eight studies evaluating the ability of gene expression analysis to correctly reproduce HER2 status either gave insufficient concordance levels or were unable to evaluate agreement between techniques [28, 32, 39–44].

Two recent large studies evaluated the concordance between the qRT-PCR assessment of HER2 transcript in the Oncotype DX assay and FISH [45, 46], and an additional study compared qRT-PCR and IHC [47]. These studies reported high concordance rates between the tests, but concluded that the concordance rate was insufficient to support the use of molecular tests instead of in situ methods, in particular when equivocal cases were included in the analysis [12].

In a recent work Noske et al. [48] observed, in a cohort of 278 patients from the GeparTrio trial, a highly significant correlation and a high overall agreement between IHC, supplemented by SISH for equivocal cases, and qRT-PCR in determining HER2 status. These authors also observed a significant discordance between centralized and local HER2 determination, hence suggesting the importance of reference laboratories for these analyses. In contrast with these authors Viale et al. in their analysis of HER2 determination by TargetPrint in the first 800 patients enrolled in the Mindact Trial, did not find a significant difference between centralized and local HER2 determination. The range of discordance was 0–4.3% for HER2 in six centers [49], likely reflecting a better training of the pathologists taking part to the trial.

Furthermore, in the last years, the College of American Pathologists (CAP) has undertaken comprehensive efforts to educate pathologists about ways to improve laboratory performance of HER2, ER, and PgR assays [1].

The critical point of this quite vast literature is that it does not consistently show a sufficient level of agreement between molecular and tissue-based tests in defining HER2 status, when judged according to ASCO/ACP criteria. Consequently, the use of PCR-based tests in place of conventional in situ methods is not recommended [12].

Technical limitations accounting for part of the discrepancies of molecular tests can be circumvented by a proper methodological standardization. The putative dilution or amplification effects are theoretically more severe because they pertain to the nature itself of molecular tests. In fact, these tests may potentially lead to false negative results, especially when a high level of intra-tumor heterogeneity accompanies gene amplification. The results of our reconstitution in vitro experiments do not support this supposed technical flaw. Both Q-PCR and qRT-PCR tests were able to detect the HER2 increased signal over in a wide range of amplified vs. not amplified cells ratios, with fractions of amplified cells as small as 5%. However, we are also aware that HER2 copy number change in the cell lines tested would have led us to different threshold values in our reconstitution experiments. For example, the sensitivity of the method would have been lower, if a low-level HER2 amplified cell line had been used in place of SKBR3. It is therefore our opinion that the current uncertainties concerning PCR-based methods are more due to lack of standardization than to an intrinsic limitation.

In our experience, three major points have to be satisfied for the standardization of molecular methods: (i) the supervision of sample selection by an experienced pathologist, to assure that a suitable amount of infiltrating tumor cells is analyzed and that in situ carcinoma, potentially overexpressing HER2 [50, 51], is not included in the specimen; (ii) a strict control of the quantity and quality of DNA/RNA extracted; (iii) the use of extensively validated PCR analysis algorithms to measure the DNA/RNA templates in the tumor samples [19].

In this study, the results obtained on the overall patient population show a high overall concordance (94.1%) in HER2 copy number, as determined by Q-PCR and FISH. Assuming FISH as the reference standard, the sensitivity and specificity of Q-PCR are 86 and 99%, respectively, with a global accuracy of 98%. These data support the possibility to use Q-PCR, as carried out in this study, as an acceptable alternative for FISH.

On the other hand, the comparison of HER2 mRNA expression, as determined by qRT-PCR, with FISH results gives an overall concordance of 90.8%, insufficient to warrant the use of gene expression analysis in place of FISH, according to 2013 ASCO/CAP criteria and to our pre-specified criteria [1].

Viale et al. in a recent large study confirm that the positive agreement for TargetPrint, a microarray analysis to determine HER2 with IHC/FISH is comparable with other mRNA readouts, implying that gene expression and DNA evaluation of HER2 may not be interchangeable [49].

Nonetheless, the comparison of results from the total population with those from the subgroup of non-equivocal cases only (Table 2) clearly shows that the disagreement between qRT-PCR and FISH or Q-PCR is essentially limited to equivocal cases, as defined by ASCO/CAP criteria for FISH assessment of HER2 status.

In fact, one-third of these patients, considered HER2− by both FISH and Q-PCR, shows some degree of HER2 mRNA overexpression by qRT-PCR analysis compared to negative controls. These discordant cases are more prevalent in the equivocal subgroup, in which HER2 copy number has a relatively small range of variation. A putative high false-positive rate of the qRT-PCR test in this range of values may offer an easy explanation for the observed results. However, previous analyses on frozen breast cancer samples made by solid matrix blotting techniques unequivocally demonstrated that gene amplification is closely associated with protein overexpression [7]. Consequently, HER2 protein direct measurement can be well considered as the true standard control for all the other surrogate methods for HER2 characterization [4].

We therefore hypothesized that the high accuracy of qRT-PCR compared to the other methods for HER2 status evaluation could account for the discordant results we observed. Accordingly, we set up a control experiment by directly measuring HER2 protein levels in a representative subgroup of cases (Table 3).

The analysis conducted directly on this case set shows a strong correlation between mRNA and protein levels. In particular, high HER2 protein levels were found in qRT-PCR positive, IHC/FISH equivocal cases, but not in those lacking mRNA overexpression. These results suggest that qRT-PCR may indeed be a better proxy for determining HER2 protein levels than FISH. Moreover, qRT-PCR may be of greater use than FISH in those cases with low level of HER2 amplification, when in situ analysis can miss its surrogate endpoint of identifying HER2 positive cases. Hence, as for Q-PCR, qRT-PCR may find its role in the subpopulation of patients for whom a reflex test is required after FISH yields equivocal results, or where the starting material is too exiguous for definitive FISH assessment (e.g., in fine needle aspiration biopsies, as we showed in a previous publication [52]). It is presently unclear which of the two molecular methods should be preferred.

Indeed, in light of the above considerations, qRT-PCR also outperforms Q-PCR in specific settings, our conclusion apparently contradicts the results of in vitro serial dilution experiments showing an equal detection threshold of the two molecular tests. However, it should be noted that these experiments were designed to address the question of the dilution effect, and not to determine the minimal amount of HER2 DNA or RNA molecules respectively detectable by the two PCR-based assays.

High levels of HER2 protein were also found in the two non-equivocal cases with mRNA overexpression without amplification according to both FISH and copy number analysis. Cases like these could represent a true alteration of gene expression control or alternatively a very low level of amplification [2, 53]. The most relevant point in such cases is that they reinforce the notion of the superior accuracy of qRT-PCR compared to other methods to define HER2 status. However, we feel the need to caution the Reader about the possible over-interpretation of our results, in that the case set for which we could perform the combined assessment of DNA copy number by both FISH and Q-PCR, RNA abundance, and protein levels is relatively small, hence the results deriving from its analysis are to be considered as exploratory and hypothesis-generating rather than conclusive. Another relevant point to be taken into consideration is that in our series, the optimal cut-off for defining the results of q-RT-PCR as positive was obtained by fitting a model. Were this method to be diffused amongst laboratories, a universal calibration reference should be used (e.g., cDNA from a known and well-studied breast cancer HER2-amplified cell line mixed at serial dilutions with donors’ cDNA, with the caveat of using the same batch of cancer cell lines from a commercial vendor due to the risk of copy number drift over time for cell cultures kept in individual laboratories). A promising solution to such non-trivial limitation would be the adoption of digital PCR, which does not require normalization with reference transcripts. Reports in this regard have been recently published [54–56], and prospective studies should aim to assess the role of such novel and potentially more accurate methods in the landscape of HER2 status determination technologies.

While not meaning to be exhaustive (which would be outside the scope of the present article), we wish to observe that the costs of PCR methods are, in general, extremely affordable: the acquisition of a good thermocycler in the European Community is in the order of few tens of thousand euros for top brands, and the actual cost (with the reagents described in the “Methods”) of a PCR performed in technical triplicate is around 30 Euro (33 US $ at the current conversion rate). Moreover, the person-time with the help of automated nucleic acid extraction devices is relatively contained, generally less than 1 h and a half of hands-on time and data analysis. These layman considerations should be taken into account for possible practical uses of PCR in HER2 determination in real-life practice and in the proper context (i.e., when skilled personnel is available).

Grant et al. supported the use of microarray analysis by TargetPrint to determine HER2 status in breast cancer. This study found a 100% concordance rate between IHC/FISH and TargetPrint results for HER2 tumour status and, furthermore, added important informations in all equivocal cases of study. HER2 positive status assigned by TargetPrint in those patients with an equivocal ISH result facilitated treatment decision-making based on a binary value, which excluded the previous uncertainty of indeterminate ISH reporting [57].

From a clinical point of view, patients with HER2 transcript and protein overexpression, with a negative FISH result, are considered HER2− according to standard criteria, and consequently they are not eligible for anti-HER2 therapies.

In our series these cases were around 8%, although this value is not representative of the entire breast cancer population, because we deliberately enriched our sample set in equivocal cases. These cases are likely to be more rarely observed in daily practice, albeit, extrapolating from our study and from breast cancer epidemiology, they may represent a small but clinically relevant breast cancer population not benefitting from anti-HER2 treatments.

These cases we characterized might well correspond to the small breast cancer patients subset considered as HER2-tumors but, nonetheless, responding to trastuzumab in both adjuvant and metastatic settings [7, 8, 10]. Breast cancer samples overexpressing HER2 in the absence of gene amplification can also be found in the METABRIC project dataset [58]. The cause of HER2 non-amplification-driven overexpression in this small breast cancer subset is unclear, although aberrant regulation by miRNAs [59, 60] may play a role in this context.

## Conclusion

While pre-analytical limitations can affect both PCR and FISH, IHC and, especially, ISH analysis are prone to subjective evaluation even by skilled operators: our work suggests that qRT-PCR could be proposed as a more objective test, especially in FISH equivocal cases. Potential applications include the use of qRT-PCR as a second level reflex test after FISH, and the determination of HER2 status when analytical material is too little for obtaining an accurate result, such as in certain fine needle aspiration biopsies.

From a biological perspective, the results of our qRT-PCR analyses provide the proof of principle that this technique may be as accurate as FISH or even more in certain contexts to identify patients with HER2 protein increased abundance.

Furthermore, our analyses support the use of qRT-PCR for HER2 mRNA expression as a second level test to define HER2 status in FISH equivocal cases, in order to administer potentially effective anti-HER2 treatments in this small but non-negligible subset of patients.

In the future, PCR methods should achieve a greater standardization, and being faster and cheaper than other methods to detect HER2 on DNA and RNA levels, they could be useful adjuncts of IHC/FISH. Prospective assessments in larger patient populations are needed for a more exhaustive resolution of this relevant issue.

## Abbreviations

ASCO/CAP: American Society of Clinical Oncology/College of American Pathologists
AOU: Azienda ospedaliera-universitaria
BC: breast cancer
CISH: chromogenic in situ hybridization
FISH: fluorescent-in situ hybridization
HER2: human epidermal growth factor receptor 2
IHC: immunohistochemistry
IRCCS: Istituto di ricovero e cura a carattere scientifico
ISH: in situ hybridization
IST: Istituto Tumori
NA: negative agreement
OA: overall agreement
PA: positive agreement
qPCR: quantitative polymerase-chain-reaction
qRT-PCR: real-time quantitative polymerase-chain-reaction
